# Is There an Epigenetic Component Underlying the Resistance of Triple-Negative Breast Cancers to Parp Inhibitors?

**DOI:** 10.3389/fphar.2012.00202

**Published:** 2012-12-27

**Authors:** Amanda Lovato, Lawrence Panasci, Michael Witcher

**Affiliations:** ^1^The Departments of Oncology and Experimental Medicine, The Lady Davis Institute and Segal Cancer Centre of the Jewish General Hospital, McGill University MontrealQC, Canada

**Keywords:** Parp inhibitors, epigenetics, breast cancer, transcription factors, therapeutic resistance

## Abstract

Poly(ADP-ribose) polymerase (Parp) is an enzyme responsible for catalyzing post-translational modifications through the addition of poly(ADP-ribose) chains (known as PARylation). Modification by PARylation modulates numerous cellular processes including transcription, chromatin remodeling, apoptosis, and DNA damage repair. In particular, the role of Parp activation in response to DNA damage has been intensely studied. Tumors bearing mutations of the breast cancer susceptibility genes, Brca1/2, are prone to DNA breakages whose restoration into functional double-strand DNA is Parp dependent. This concept has been exploited therapeutically in Brca mutated breast and ovarian tumors, where acute sensitivity to Parp inhibitors is observed. Based on *in vitro* and clinical studies it remains unclear to what extent Parp inhibitors can be utilized beyond treating Brca mutated tumors. This review will focus on the often overlooked roles of PARylation in chromatin remodeling, epigenetics, and transcription to explain why some cancers may be unresponsive to Parp inhibition. We predict that understanding the impact of PARylation on gene expression will lead to alternative approaches to manipulate the Parp pathway for therapeutic benefit.

## The Clinical Landscape of Parp Inhibitors as Anti-Cancer Therapeutics

Breast cancer is an epidemic afflicting approximately one out of every nine women (Kurian et al., 2010). This heterogeneous disease is clinically stratified into four major subtypes; luminal A, luminal B, Her2+ [elevated Her2, Estrogen receptor (ER) negative], and triple-negative (Her2, ER, and progesterone receptor negative). However, current literature indicates further stratification using additional clinical markers, such as cytokeratins, Cyclin D1, and Claudin (Gusterson et al., 2005; Prat et al., 2010; Curtis et al., 2012), will provide more precise prognostic and predictive information. Patients whose tumors fall into the triple-negative category have the poorest clinical outcomes. While these aggressive tumors initially respond to chemotherapy, triple-negative patients are at high risk for metastatic recurrence and have poor overall survival (Di Cosimo and Baselga, 2010). Clearly, new therapeutic strategies are desperately needed to combat triple-negative tumors both at the time of onset and if necessary, at recurrence.

Approximately 10–20% of triple-negative tumors harbor a mutation in the *Brca1/2* genes (Gonzalez-Angulo et al., 2011; Pern et al., 2012; Phuah et al., 2012). Brca1/2 proteins are an integral part of the homologous-recombination (HR) mediated DNA damage repair process. When mutated, the repair of double-strand breaks by HR is compromised. Mutations within the *Brca* genes are known to confer a 50–80% lifetime risk of breast cancer and a 20–40% lifetime risk of ovarian cancer for female carriers (Ford et al., 1998). An exciting new development in the field of cancer therapy is the exploitation of this defect using Poly(ADP-ribose) polymerase (Parp) inhibitors to generate synthetic lethality. Parp inhibitors have been used successfully to achieve clinical responses in patients with Brca mutated breast or ovarian cancers (Fong et al., 2009; Audeh et al., 2010; Tutt et al., 2010). Notably, the adverse effects of Parp inhibitors seen in clinical trials are quite mild with few signs that non-tumorigenic tissue is targeted by these drugs.

The role of Parp in the DNA damage repair response is multifaceted and already well-reviewed (Aly and Ganesan, 2011; Helleday, 2011). It is generally suggested throughout the scientific literature that Parp inhibitors generate an accumulation of single-strand DNA breaks in Brca mutated cells, which are subsequently processed to double-strand breaks during replication. However, the precise mechanism whereby Brca mutations and Parp inhibition combine to create a synthetic lethal effect remains somewhat controversial, with multiple mechanisms proposed (Helleday, 2011). Nonetheless, it seems clear that Parp inhibitors lead to stalled replication forks and an accumulation of DNA damage, particularly cytotoxic in cells with a mutant Brca background.

The clinical efficacy of Parp inhibitors in triple-negative breast tumors bearing Brca mutations has sparked interest to initiate new clinical trials in triple-negative patients having wild-type Brca with the expectation that combining chemotherapy with Parp inhibitors will enhance the outcomes of currently utilized therapies. While the results from a few phase one trials are encouraging, to date, most have been met with limited success. Preliminary data from a cohort of 86 triple-negative patients co-treated with the Parp inhibitor Bsi-201 (iniparib) and gemcitabine showed improved overall survival (O’Shaughnessy et al., 2009). Similarly, co-treatment of a 123 triple-negative patient cohort with gemcitabine and iniparib improved overall survival from 7.7 to 12.3 months (O’Shaughnessy et al., 2011). But a larger phase III trial involving 519 women showed co-treatment of cytotoxic agents with the Parp inhibitor Iniparib was associated with disease progression in most cases (O’Shaughnessy, 2011). However, results from these trials should be taken with caution as recent data suggests Parp is not the primary target of iniparib (Liu et al., 2012). Another phase II trial looking at the efficacy of the Parp inhibitor olaparib in breast cancer patients failed to show significant clinical responses (Gelmon et al., 2011). Encouragingly, in this same study, a cohort of patients with ovarian cancers did demonstrate partial responses to olaparib regardless of Brca status.

Overall, the early indicators from trials involving Parp inhibitors for triple-negative breast cancer show partial, but not complete responses. Encouragingly, there are clearly patients who do respond to these therapies. Recent research has identified a subset of triple-negative tumors that have an increased likelihood to respond to Parp inhibition. Defective proteins in the HR repair system, or epigenetically silenced Brca, amongst other defects, contribute to a molecular pathology that is not unlike tumors bearing Brca mutations. These tumor properties are defined as having “Brcaness” or being “Brca-like” (Turner et al., 2004; Ratner et al., 2012). This concept has led to the hypothesis that tumors with features of Brcaness may respond to Parp inhibition. The concept of Brcaness has been used in retrospective study to predict response to platinum-based therapies in 8 of 10 patients (Konstantinopoulos et al., 2010). But a recent study analyzing data from 101 patients receiving adjuvant cyclophosphamide-based chemotherapy showed Brcaness could not predict differences in patient survival (Oonk et al., 2012). However, it still remains to be determined if pathological features of Brcaness may be a more powerful predictor of sensitivity to Parp inhibitors than conventional chemotherapy.

Current literature suggests several hypotheses to predict sensitivity to Parp inhibition in subsets of triple-negative tumors, but there is a lack of evidence from *in vitro* and mouse studies suggesting that established triple-negative cell lines are sensitive to clinically relevant Parp inhibitors or Parp-1 knock down. In fact, unpublished data from our lab and another recent report show very high concentrations of commonly used Parp inhibitors are needed to suppress the growth of triple-negative cell lines *in vitro* (Chuang et al., 2012). The micromolar concentrations of inhibitors needed to suppress proliferation is likely well beyond those required to block Parp activity (Bryant et al., 2005; Liu et al., 2008), and may reflect secondary effects of these inhibitors.

**Figure 1:**
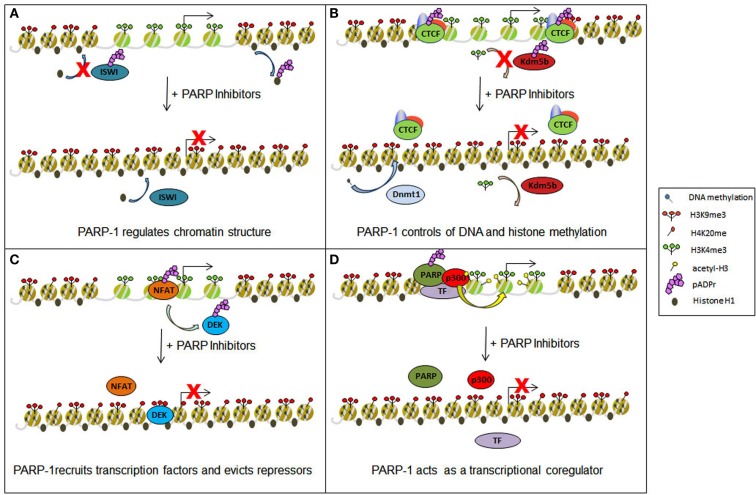
**Epigenetic and transcriptional impact of Parp inhibitors**. Parp inhibitors may contribute to epigenetic and transcriptional deregulation in cells through several different mechanisms. **(A)** The drug-induced spread of heterochromatin may result from the release of the protein Iswi from inhibition, promoting histone H1 integration into chromatin, and through the prevention of histone H1 removal from chromatin by direct PARylation. **(B)** Chromatin boundaries normally maintained by PARylated Ctcf may be disrupted and demethylation of H3K4me3 by Kdm5b restored with the use of Parp inhibitors. In both incidencies, transcriptional inhibition will ensue. Such drugs may also act to restore Dnmt1 methylation of DNA, further promoting gene silencing. **(C)** Parp inhibition can disrupt protein: DNA interactions, preventing the maintenance of certain trans-activating factors (e.g., Nfat) at transcription start sites while also causing the retention of some repressor proteins (e.g., Dek). **(D)** Gene activation may also be negatively regulated by preventing Parp from acting as a transcriptional coregulator and obstructing the recruitment of such proteins as the histone acetyl transferase p300.

Beyond breast cancer, early phase clinical trials with Parp inhibitors in combination with standard chemotherapy have been met with either partial responses, or a lack of clinically relevant responses in multiple types of solid tumors (Plummer et al., 2008; Khan et al., 2011; Kummar et al., 2011, 2012; Rajan et al., 2012). We propose that activities of Parp, being targeted by inhibitors beyond those in the DNA damage repair process, account for their limited success in a wild-type Brca background (Figure 1). Specifically, we hypothesize that targeting Parp will impair its role in regulating the expression of tumor suppressor genes, thereby generating unwanted consequences. However, we further propose that understanding the mechanisms whereby Parp activates transcription may be used to predict new, more potent therapeutic approaches.

## Poly(ADP)Polymerases, More than DNA Repair Enzymes

The Parp family of enzymes encompasses multiple proteins (up to 17) of varying degrees of homology (with the main conservation residing in the Parp catalytic domain), all of whom use NAD+ as a substrate to catalyze the addition of ADP-ribose moieties onto target proteins (Kim et al., 2005). Among the Parp proteins, only Parp-1 and Parp-2 build “poly” ADP-ribose polymers. The other family members, including Parp-3, are capable of adding only a monomeric ADP-ribose to proteins. Of Parp-1 and 2, Parp-1 has been more extensively studied and will therefore be the focus of this review. Under basal conditions, Parp-1 is active, but modifies relatively few target proteins compared to conditions of cellular stress and after DNA damage (Gagne et al., 2008, 2012; Witcher and Emerson, 2009). Parp-1 recognizes and binds specific DNA secondary structures commonly associated with damaged DNA including single-strand DNA, double-strand breaks, and crossovers. Upon binding, its enzymatic activity is allosterically triggered (D’Amours et al., 1999; Kun et al., 2002). This particular mechanism of regulation allows localized activation of Parp-1 for targeted repair of DNA damage. However, numerous studies have also demonstrated that Parp-1 is recruited to chromatin, which acts as an on-switch for its enzymatic activity in the absence of DNA damaging agents (Poirier et al., 1982; Ding and Smulson, 1994; Kim et al., 2004; Lonskaya et al., 2005; Pinnola et al., 2007; Wacker et al., 2007).

Parp-1 associates with the chromatin of promoter regions in a significant proportion of actively transcribed genes throughout the genome (Krishnakumar and Kraus, 2010; Zhang et al., 2012) and copurifies biochemically with RNA Pol II (Slattery et al., 1983). Consistent with this, multiple studies utilizing Parp-1 depleted cells have substantiated an activating role for Parp-1 in gene regulation (Ziegler and Oei, 2001; Ogino et al., 2007; Krishnakumar et al., 2008; Okada et al., 2008; Frizzell et al., 2009).

How does Parp modulate gene expression? It does so through a multi-pronged approach, with individual actions cooperating to fine tune the transcriptional process (Figure 1). Parp-1 regulates transcription minimally through (1) the alteration of chromatin structure (2) the control of DNA and histone methylation status (3) the recruitment and maintenance of transcription factors to promoter regions, and (4) acting as a transcriptional coregulator.

## Parp-1 as a Regulator of Transcription

First, direct PARylation of histones can lead to a loosening of chromatin conformation. PARylation of bulk nucleosomes *in vitro* leads to decondensation (Faraone-Mennella et al., 1993) and Parp activity is required for chromatin loosening at stress induced genes in *Drosophila* (Tulin and Spradling, 2003). Biochemically, the negative charge conferred by the PAR group onto histones promotes their release from the DNA due to charge repulsion. Such is the case for histone H1, a heterochromatin-promoting factor whose PARylation-dependent removal from chromatin serves to promote chromatin relaxation (Poirier et al., 1982; Huletsky et al., 1989). Interestingly, Parp-1 localization is inversely related to histone H1 binding throughout the genome and higher proportions of Parp-1:H1 proteins tend to indicate active promoters (Krishnakumar et al., 2008). Consistent with this, experiments using Parp-1 mutants and Parp inhibitors in *Drosophila* revealed more pronounced heterochromatin at the Heat shock protein 70 (Hsp70) locus (Tulin and Spradling, 2003). Likewise, an RNAi screen revealed Parp is necessary for nucleosome eviction from chromatin at the Hsp70 locus during the rapid gene induction response to heat shock (Petesch and Lis, 2008). Recently, it has been demonstrated that lipopolysaccharide-induced Parp activity displaces nucleosomes from target genes, thereby facilitating transcription, as trans-activating factors will not have to contend with the physical obstacle of dense nucleosomes at these promoters (Martinez-Zamudio and Ha, 2012). In sum, the ability of Parp-1 to remodel chromatin in a manner conducive to transcriptional activation strongly suggests that Parp-1 primarily acts as a potent activator of transcription. However, in some contexts, Parp-1 may promote a repressed chromatin conformation when enzymatically inactive, and a more loose structure upon activation (Wacker et al., 2007). At a subset of promoters, under unstimulated conditions, Parp-1 presents itself in a corepressor complex with nucleolin, nucleophosmin, and Hsp70. These repressive factors, however, are released upon signal activation of Parp-1, thus providing a mechanism for differential effects of Parp-1 on chromatin structure (Ju and Rosenfeld, 2006).

Countering the effects of PARylation on chromatin structure is poly(ADP-ribose) glycohydrolase (known as Parg). Parg catabolizes ADP-ribose polymers synthesized by Parp-1. This enzymatic activity has been demonstrated to impair Parp-mediated chromatin remodeling *in vitro* (Kim et al., 2004). Chromatin remodeling mediated by Parp-1 potentiates transcriptional activation by the ER (Kim et al., 2004) and Parg was shown to suppress estrogen-dependent transcription through blocking Parp activity. Further work is required to elucidate the *in vivo* action of Parg on chromatin structure.

In addition to histone H1 removal, Parp-1 configures chromatin through modification of proteins involved in remodeling and organizing chromatin structure. PARylation generally results in protein activation, but can also result in functional suppression of chromatin remodelers. For example, PARylation is inhibitory to the function of the repressive remodeling complex Iswi (Sala et al., 2008). Iswi is known to promote the association between H1 and DNA (Corona et al., 2007), thus illustrating a complementary mechanism by which PARylation results in a reduction of H1 binding to DNA.

Notably, Parp interaction with Brg-1 (SmarcA4), together with histone deacetylases (Hdacs), results in a repressive complex, inactivating the transcription of genes involved in cardiomyocyte differentiation through deacetylation of histones (Hang et al., 2010). Thus, in particular contexts, Parp-1 activity can relay transcriptionally repressive signals. Conversely, this same study showed that a Parp-1/Brg-1 complex devoid of Hdac could activate a separate subset of genes. It remains to be seen if the PARylation of Brg-1 leads to gene activation in other tissue types.

In addition, other chromatin remodelers are modified by Parp-1 under conditions of cellular stress (Gagne et al., 2008, 2012). Proteomic studies have shown these to include TopoIIα, Brg-1, TopoIIβ, HmgA1, Chd1, Chd5, and Snf2L1. Clearly, Parp activation relays a signal that is having a profound effect on chromatin structure. However, the precise impact PARylation has on these proteins, and the subsequent effects on transcription of their target genes remains unknown.

Second, Parp activity regulates gene expression through control of epigenetic mechanisms including histone modification and DNA methylation. Parp covalently modifies the epigenetic regulatory protein CCCTC binding factor (Ctcf; Yu et al., 2004). Ctcf PARylation is important for its insulator activity, which functions to prevent enhancers and repressors from acting on distal promoters over long distances. Therefore, inhibition of Ctcf PARylation will result in altered regulation of target genes through the aberrant actions of distal regulatory regions.

Chromatin immunoprecipitation experiments and knockdown studies indicate Ctcf plays an import role in the maintenance of chromatin boundaries (Cuddapah et al., 2009; Witcher and Emerson, 2009). Repressive heterochromatin is the default state and, unless constrained, will spread passively throughout a chromosome (Talbert and Henikoff, 2006). Chromatin boundaries form a barrier prohibiting the spread of repressive chromatin. Interestingly, Ctcf PARylation has been linked to the maintenance of chromatin boundaries at tumor suppressor genes (Witcher and Emerson, 2009; Farrar et al., 2010).

This is supported by unpublished data from our lab showing Parp inhibitors lead to an accumulation of repressive histone modifications, such as H3K27me^3^, at tumor suppressor genes. We have also published that Parp-1 inhibition through knockdown, or pharmacologic approaches, results in the transcriptional repression of the *Rassf1a* and *p16* tumor suppressor genes (Witcher and Emerson, 2009). Based on this data, it is not unexpected that Parp inhibitors have been found to have transcriptionally repressive effects on tumor suppressor genes (Witcher and Emerson, 2009; Nguyen et al., 2011). However, it remains to be proven that these negative effects are mediated by disruption of Ctcf function.

Beyond modulating its role as a chromatin boundary protein, PARylation of Ctcf may act as a docking site for Dnmt1 binding (Zampieri et al., 2009, 2012). This interaction is thought to act a molecular sponge, prohibiting Dnmt1 from methylating regions surrounding Ctcf binding sites. Elegant studies utilizing engineered mutations of endogenous Ctcf sites clearly show localized accumulation of DNA methylation when Ctcf binding is abolished (Pant et al., 2004; Davalos-Salas et al., 2011). However, it remains to be proven that the interaction between Ctcf and Dnmt1 is pivotal for the capacity of Ctcf to prevent DNA methylation. Nevertheless, this model again suggests that a loss of Ctcf PARylation brought about by Parp inhibitors would result in profound epigenetic changes and significant changes to gene expression throughout the genome. Supporting this model is at least one study showing that Parp inhibition does indeed result in widespread accumulation of DNA methylation (Reale et al., 2005).

In addition to epigenetic regulation through Ctcf, PARylation of other target proteins organize chromatin structure through coordinating the placement of histone modifications. This has been most clearly demonstrated for the histone demethylase Kdm5b (Krishnakumar and Kraus, 2010). Modification of Kdm5b by Parp-1 allows H3K4me3 to persist in the promoter regions of actively transcribed genes. H3K4me3 is important for loading the PolII machinery at most, if not all, actively transcribed genes. PARylation of Kdm5b prevents active demethylation of this mark at Kdm5b target genes, thus promoting transcription. Knockdown of Parp-1 potently blocks transcription of Kdm5b target genes through this mechanism. Clearly, small molecule inhibitors or Parp-1 would be expected to disrupt transcription in a similar fashion.

Third, Parp activity controls the recruitment and maintenance of transcription factors to promoter regions. As stated above, classic experiments from the Roder lab demonstrate that Parpcopurifies biochemically with RNA PolII (Slattery et al., 1983). Follow up work showed Parp enhances the assembly of the preinitiation complex *in vitro* (Meisterernst et al., 1997). Surprisingly, Parp activity has been demonstrated to be necessary to retain Pol II at actively transcribed target genes (Zobeck et al., 2010). It is postulated that PAR polymers creates a scaffold that retains Pol II at gene loci. It is quite possible, but remains to be proven, that PAR scaffolds act to retain transactivators at target genes as well. The trans-activating factors Nfat, Klf8, and Tef-1 are associated with, and activated by PAR polymers (Butler and Ordahl, 1999; Olabisi et al., 2008; Lu et al., 2011). It is possible the mechanism lies in the retention of these factors at target genes by (ADP)ribose polymers.

In contrast to this model, PARylation of the transcriptional repressor Dek by Parp-1 serves to evict Dek from chromatin, ultimately promoting gene activation (Gamble and Fisher, 2007). Thus, blocking the actions of repressors serves as another mechanism through which Parp activates transcription.

Fourth, Parp-1 itself has also been described to act as a coregulator of transcription. It is recruited to genes via interaction with DNA binding factors. As a coregulator, Parp-1 can be integrated into complexes having stimulatory effects on transcription mediated by transcription factors such as NF-κβ and AP-2 (Li et al., 2004). To complete the assembly of the NF-κβ activating complex, Parp-1 is required for the integration of the histone acetyltransferase p300 (Hassa et al., 2003; Kaur et al., 2006), providing another link between Parp-1 and histone modification. Parp-1 is also a component of a coactivating complex responsible for driving nuclear hormone receptor-mediated transcription in response to estrogens and retinoids (Pavri et al., 2005; Ju et al., 2006).

Docking with Parp-1 can also serve to enhanced phosphorylation of the associated transcription factor leading to heightened trans-activating capabilities. This has been demonstrated for B-Myb in a cell cycle dependent fashion (Cervellera and Sala, 2000; Santilli et al., 2001) and Elk-1 in response to Erk-1 activation (Cohen-Armon et al., 2007). These data indicate Parp activity links intra and extracellular signaling events with gene induction.

Parp-1 knockdown studies show Parp activity also functions to repress a subset of genes (Frizzell et al., 2009). Consistent with this, PARylation of a number of transcription factors has been described to prevent their interaction with DNA. This has been described for Smads (after TGFβ stimulation; Lonn et al., 2010), p53, and Sp1 (Kumari et al., 1998; Malanga et al., 1998; Zaniolo et al., 2007). Inactivation of Smads by Parp following TGFβ signaling remains controversial as a more recent study found Parp necessary for Smad activation post-TGFβ exposure (Huang et al., 2011). It will be of great interest in the future to determine how Parp integrates signaling events such as TGFβ, reactive oxygen species, and growth factors into coordinated transcriptional outputs.

Information from *in vitro* studies describing the inhibition of transcription factor binding to cognate sites by PARylation should be taken with caution. Without the constraints found *in vivo*, ADP-ribose polymers can be extended to enormous lengths *in vitro* (D’Amours et al., 1999; Mendoza-Alvarez et al., 2000). It is likely that the formation of such a network of polymers could impair binding to cognate DNA sites *in vitro* due to simple steric hindrance.

Overall, evidence suggests that Parp-1 plays primarily a stimulatory role on transcription, including activation of tumor suppressor genes and a more minor role in gene repression. Clearly there is overwhelming data demonstrating that Parp-1 participates in gene regulation at multiple levels, most prominently by coordinating transcription factor activity and organizing chromatin structure. It is imperative that these aspects of Parp-1 function be considered, along with its role in DNA damage repair if we are to extend the clinical use of Parp inhibitors to treat tumors beyond those bearing Brca mutations.

## Clinical Modulation of the Poly(ADP)Ribose Pathway: Future Perspectives

The epigenetic, chromatin remodeling, and transcriptional regulatory roles of Parp-1 are necessary to activate a group of genes under basal conditions and another cohort in response to stimuli, such as cell stress. Therefore, inhibiting Parp-1 will potentially disrupt expression of a wide range of genes, including tumor suppressor genes, which may limit the benefits of Parp inhibitors in Brca wild-type patients.

That being said, can this pathway be targeted successfully to treat a broad range of tumors? In our opinion, yes, but we need to revisit our approach. First, it is clear that Parp-2 can compensate for Parp-1. Thus Parp inhibitors should be tested for their capacity to block the activity of both proteins before being considered for clinical trials. Such consideration might have prevented the failed clinical trials with iniparib, a drug initially described as a Parp inhibitor, but that has recently been proven to lack such activity (Patel et al., 2012).

Second, the concept of Brcaness needs to be more clearly defined using both genetic and epigenetic markers. It is probable that tumors with epigenetically silenced, as well as mutated, DNA repair genes will be sensitive to Parp inhibitors. This concept may also be employed to predict successful combinations of chemotherapeutics with Parp inhibitiors. Further, it is possible that epigenetic silencing of tumor suppressor genes that are Parp targets may negate any pro-proliferative effects of Parp, potentially rendering the cells sensitive to Parp inhibition. To date, *in vitro* models have been used to accurately predict tumor pathologies that are clinically sensitive to Parp inhibitors (Bryant et al., 2005; Donawho et al., 2007). Therefore, future testing of Brcaness models using *in vitro* systems will be an important stepping stone to make these models clinically relevant.

**Figure 2:**
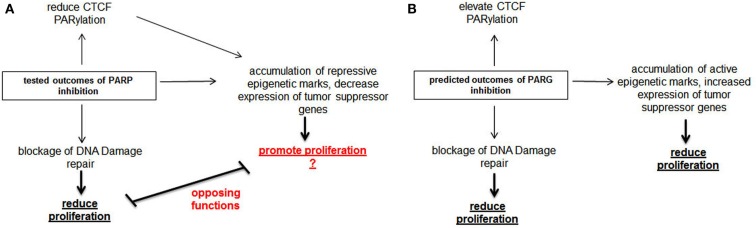
**Comparison of molecular action of Parp inhibitors with predicted action of Parg inhibitors**. **(A)** Parp inhibitors inhibit the growth of Brca mutated tumors through blocking DNA damage repair. Parp inhibitors also dePARylate Ctcf, cause the accumulation of repressive epigenetic marks at tumor suppressor genes and diminish the expression of these genes. We propose that these properties of Parp inhibitors will limit their usefulness as anti-cancer therapeutics. **(B)** In contrast, we predict Parg inhibitors will stimulate Ctcf PARylation and the transcription of tumor suppressor genes, in addition to blocking the DNA damage repair response. Thus, Parg inhibition may have greater potential as an anti-cancer therapeutic than Parp inhibitors.

Finally, we propose that Parg represents an attractive therapeutic target (Figure 2). The understudied protein Parg catalyzes the counter-activity of Parp-1/2 by removing PARylation modifications from target proteins. Intriguingly, Parg has a critical role in DNA damage repair, similar to Parp-1 (Fisher et al., 2007; Mortusewicz et al., 2011). Counter intuitively, while PARylation of target proteins is necessary to initiate the repair response to DNA damage, removal of these tags is essential for a complete DNA damage response. Thus, Parg inhibition would likely create a synthetic lethal situation in Brca defective cells in a similar manner as Parp inhibitors. Consistent with this concept, Brca mutated cells have been shown to be highly sensitive to an inhibitor of Parg (Fathers et al., 2012). Further, we now have exciting new data demonstrating Parg inhibition is a relevant approach to inhibit the proliferation of triple-negative breast cancer cell both *in vitro* and *in vivo* (manuscript in preparation).

Parp-1 is an important activator of transcription and probably plays an important role in promoting transcription of tumor suppress genes. Therefore, we postulate Parp inhibitors may actually have pro-oncogenic effects on some cell populations. But, what is the impact of inhibiting Parg on these same processes? While the overall impact of Parg on gene regulation remains unclear at this time, it has been shown that Parg can block Parp-1 mediated chromatin remodeling and transcriptional activation in specific circumstances. Therefore, we speculate Parg inhibition might heighten the effects of PARylation, thus promoting the transcription of tumor suppressor genes. Further, in cancer cells having defects in the PARylation pathway such as aberrantly dePARylated Ctcf, Parg inhibition might serve to correct these deficiencies.

Supporting these rationale for Parg inhibition being a novel approach for anti-cancer therapy are several reports indicating Parg inhibition has potent anti-tumor effects against cholangiocarinoma *in vivo* (Marienfeld et al., 2003), and *in vitro* data showing growth inhibitory activity of Parg inhibition or knockdown in multiple types of cancer (Fauzee et al., 2012; Feng et al., 2012; Li et al., 2012; Pan et al., 2012). Now that the crystal structure of Parg has been solved (Slade et al., 2011; Kim et al., 2012), there is a clear need to develop specific inhibitors of this enzyme and test their efficacy as anti-cancer therapeutics.

## Conflict of Interest Statement

The authors declare that the research was conducted in the absence of any commercial or financial relationships that could be construed as a potential conflict of interest.
